# Correction: Additional outreach effort of providing an opportunity to obtain a kit for fecal immunochemical test during the general health check-up to improve colorectal cancer screening rate in Japan: A longitudinal study

**DOI:** 10.1371/journal.pone.0339049

**Published:** 2025-12-15

**Authors:** The PLOS One Staff

The corresponding author’s email address is incorrect. Misuzu Fujita’s email address is: mi-hujita@kenko-chiba.or.jp.

The publisher apologizes for the error.
